# Surface Characterization and Conductivity of Two Types of Lithium-Based Glass Ceramics after Accelerating Ageing

**DOI:** 10.3390/ma13245632

**Published:** 2020-12-10

**Authors:** Marko Jakovac, Teodoro Klaser, Borna Radatović, Željko Skoko, Luka Pavić, Mark Žic

**Affiliations:** 1 Department of Prosthodontics, School of Dental Medicine, University Zagreb, 10000 Zagreb, Croatia; jakovac@sfzg.hr; 2 Department of Physics, Faculty of Science, University of Zagreb, 10000 Zagreb, Croatia; tklaser@phy.hr (T.K.); zskoko@phy.hr (Ž.S.); 3 Center for Excellence for Advanced Materials and Sensing Devices, Institute of Physics, 10000 Zagreb, Croatia; bradatovic@ifs.hr; 4 Ruđer Bošković Institute, P.O. Box 180, 10000 Zagreb, Croatia; Luka.Pavic@irb.hr

**Keywords:** glass ceramics, lithium disilicate, zirconia-reinforced lithium silicate, degradation, ageing, impedance, conductivity, XRPD, SEM, acetic acid

## Abstract

In this study, two different dental ceramics, based on zirconia-reinforced lithium-silicate (LS1) glass-ceramics (Celtra Duo, Dentsply Sirona, Bensheim, Germany) and lithium disilicate (LS2) ceramics (IPS e.max CAD, Ivoclar, Vivadent, Schaan, Liechtenstein) were examined. They were tested prior to and after the crystallization by sintering in the dental furnace. Additionally, the impact of ageing on ceramic degradability was investigated by immersing it in 4% acetic acid at 80 °C for 16 h. The degradability of the materials was monitored by Impedance Spectroscopy (IS), X-Ray Powder Diffraction (XRPD), and Field Emission Scanning Electron Microscope (FE-SEM) techniques. It was detected that LS2 (vs. LS1) samples had a lower conductivity, which can be explained by reduced portions of structural defects. XRPD analyses also showed that the ageing increased the portion of defects in ceramics, which facilitated the ion diffusion and degradation of samples. To summarize, this study suggests that the non-destructive IS technique can be employed to probe the ageing properties of the investigated LS1 and LS2 ceramics materials.

## 1. Introduction

Nowadays, progress in both dental material production and computer engineering has yielded a rapid development in computer-aided design/computer-aided manufacturing (CAD/CAM) technology [1,2]. For the past 35 years of the application in dentistry, this technology has evolved and is now utilized for more complex oral rehabilitation tasks [3,4,5,6]. Although it was initially designed for single unit indirect restorations, today it has reached its potential as it can be applied to even perform full-arch restorations with a high degree of accuracy and precision.

Furthermore, the most crucial parts of digital dentistry are diverse dental materials (for milling and printing) that are based on numerous composite/acrylic, metal or ceramic materials. Momentarily, a wide array of numerous dental composites that can be pre-processed in various ways prior to their application are available on the market [7,8,9,10]. However, it is crucial to determine which pre-treatments should be applied, as this has a direct impact on the materials’ durability and stability.

The most widely utilized types of dental ceramics are based on feldspar minerals [11]. Feldspathic ceramic is a natural ceramic and is mostly used for veneering metal or zirconium-oxide frameworks due to its low mechanical properties. However, for all-ceramic options, synthetic glass ceramics with leucite crystals were introduced a few decades ago [12,13,14,15,16] as a metal framework-free alternative. Both feldspathic ceramics and glass ceramics are silica based (e.g., silicon dioxide) [17].

Currently, the most promising types of glass ceramics for the restorations are based on lithium disilicate (LS2) (e.max LS_2_, IvoclarVivadent, Schaan, Liechtenstein) [18,19,20,21]. This ceramic material exhibits good mechanical properties, as well as translucency and it provides a reliable adhesive bonding to enamel and dentin with resin cements. The indications are all ceramics indirect restorations from inlays and veneers to small spans, fixed dental prostheses (three units-except molar region). However, due to the fact that the original LS2 patent period has expired, several new ceramic brands have appeared on the market.

The majority of new brands share similar compositions in the same indication range. The substitution for lithium disilicate glass ceramic on the market is a zirconia-reinforced lithium silicate glass ceramic (LS1) (Celtra Duo, SironaDentsply, Bensheim, Germany) [22,23,24,25]. These ceramics are characterized by the same indication and similar characteristics; however, the procedural steps for digital dentistry usage (CAD/CAM) are not entirely given.

Moreover, the crystallization of the green state of the lithium disilicate glass ceramics (IPS e.max CAD, Ivoclar, Vivadent, Schaan, Liechtenstein) is mandatory. On the other hand, the manufacturer’s recommendations for the LS1 pre-processing are polishing without additional crystallization or just sintering with glazing. According to Schweitzer et al. [26] and Deniz et al. [27], the sintering procedure yielded a positive effect on LS1 fatigue failure load compared to the LS1 group without sintering and glazing. Thus, pre-processing steps prior to the LS1 application are glazing and finishing dental restorations in a dental furnace [27]. As instructions regarding LS1 and LS2 pre-processing steps differ, it is of utmost importance to investigate their impact on LS1 and LS2 ageing properties.

The term “ageing properties” is used herein when commenting on changes in material properties during a certain period of time. These properties can be monitored by different methods, e.g., by immersing dental materials in diverse acids like HCl and acetic acid [18,28], by using artificial saliva (see, e.g., [29]) or by artificial ageing in an autoclave [30,31]. The aforementioned approaches result in the accelerated ageing process (i.e., a faster material degradation); and hence, they enable a long-time prediction of dental ceramic stability [28,32]. However, depending on the acid type, it is essential to precisely choose ageing temperature and the immersing time. The most common approach to probe the stability of materials is by immersing them in 4% acetic acid at 80 °C for 16 h [28].

The aim of this work was to investigate the effect of crystallization (i.e., sintering) and ageing on the LS1 and LS2 materials’ stability, i.e., their ability to resist changes in surface, chemical composition, and crystallinity. A special interest has been placed on determining the impact of the structural defects on the LS1 and LS2 conductivity.

## 2. Materials and Methods

### 2.1. Sample Preparations

In this study, two glass ceramic materials, zirconia-reinforced lithium-silicate glass (LS1) ceramics (Celtra Duo, Sirona, Dentsply, Bensheim, Germany) and lithium disilicate (LS2) ceramics (IPS e.max CAD, Ivoclar, Vivadent, Schaan, Liechtenstein), were utilized. Four different samples were examined in this work, i.e., two as-received samples and two crystallized samples. As-received LS1 and LS2 samples with no additional treatment(s) were named LS10 and LS20, while crystallized samples were labeled LS11 and LS21. The specimens were prepared as discs of 1 mm thickness and 10 mm diameter. The discs were produced according to the manufacturer’s recommendations using a dental milling unit (Cerec, MCXL, SironaDentsply, Bensheim, Germany). The sample LS11 was treated in a dental furnace (Programat P310, IvoclarVivadent, Schaan, Liechtenstein) at 820 °C for 25 min, although the manufacturer claims that crystallization is not necessary. The sample LS21 was heated at 840 °C for 25 min according to the manufacturer’s crystallization procedure.

Each sample was tested two times with different techniques, i.e., prior to and after the ageing. The experimental setup in this work enabled us to conduct tests on the same sample (e.g., LS10 disc) in the following order: XRPD, optical microscope, FE-SEM, and IS. However, after IS, all the samples were covered in gold; and thus, they were not used in further study.

### 2.2. Ageing Procedure

All samples were aged in a polypropylene flask with 25 mL of 4% acetic acid at 80 °C for 16 h to test their stability and durability (see, [28]). After the aging process, the samples were washed with water and kept at 150 °C for 48 h to remove the excess water that could interfere with the conductivity measurements.

### 2.3. Surface Investigation Methods

FE-SEM was used to determine the microstructure and morphology of the samples before the ageing process. JSM-7000F, thermal field emission scanning electron microscope (FE-SEM) manufactured by Jeol Ltd. (Tokyo, Japan) was applied. The samples were not coated with a conductive layer. The morphology of the samples after ageing was monitored by tungsten filament SEM VEGA 3 manufactured by TESCAN Ltd (Saint Petersburg, Russia).

The optical images were made using a polarizing microscope Nikon Eclipse LV150N (Nikon, Tokyo, Japan) equipped with a hot-stage controlled by a computer and a digital camera (Optoteam OPTOCAM II). The microscope is equipped with 10× and 50× magnification objectives and the sample surface images were recorded with a total magnification of 100× with LED illumination.

### 2.4. Changes in Material Structure

The difference in the structure and the impact of the aging in 4% acetic acid on the samples’ crystallinity was monitored by X-ray powder diffraction (XRPD). The samples were studied using a Philips diffractometer model PW 1820 (Philips, Almelo, The Netherlands) with CuK⍺ radiation, having a proportional counter and a graphite monochromator (Nikon, Tokyo, Japan). The measurements were done in the Bragg–Brentano geometry, in the 2θ range of 10–70°, with a step size of 0.02° and a measuring time of 3 s per step.

### 2.5. Electrical Properties

The samples for electrical property measurements were prepared as polished disks ~1 mm thick. Gold electrodes, 6 mm in diameter, were sputtered onto both sides of the samples using an SC7620 sputter coater (Quorum Technologies Ltd., Laughton, East Sussex, UK). The samples were stored in a desiccator until the measurements were performed. The electrical properties were studied by measuring the complex impedance using an impedance analyzer (Novocontrol Alpha-AN dielectric spectrometer, Novocontrol Technologies GmbH & Co. KG, Hundsangen, Germany) over the frequency range from 0.04 Hz to 1 MHz and at the 423 K. The temperature was controlled to an accuracy of ±0.5 K.

## 3. Results and Discussion

### 3.1. SEM Study

**Figure 1:**
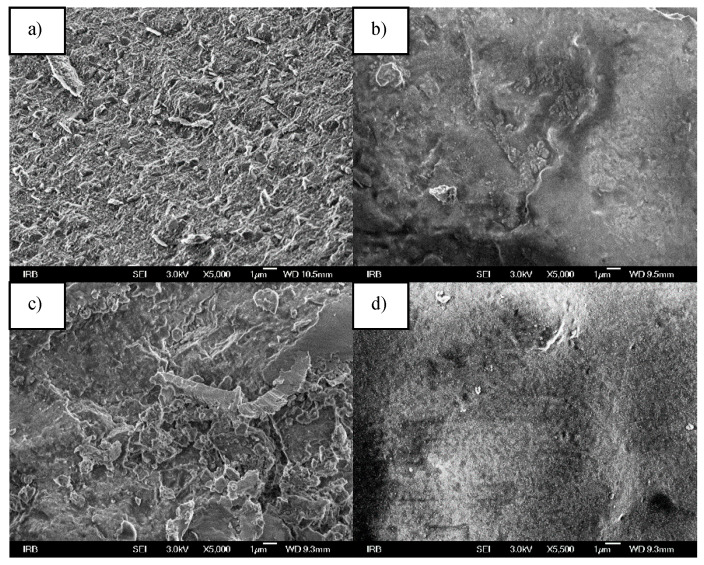
SEM images of the as-received samples (**a**) LS10, (**b**) LS11, (**c**) LS20, and (**d**) LS21. The crystallization effect can be observed in (**b**) and (**d**). Magnification is ×5000.

Figure 1 shows SEM images of the investigated non-aged LS10, LS11, LS20, and LS21 samples. As-received LS10 and LS20 samples (Figure 1a,c) show more diverse and porous morphologies that are full of grains, tubes, and fibers. On the other hand, the crystallized LS11 and LS21 (Figure 1b,d) are characterized by more uniform and less porous morphologies, which are free of surface defects (i.e., grains, tubes and fibers). Such surface defects might perturb interactions between the clinical restoration and the biological environment [28]. Moreover, the presented variations in the morphologies also suggest that the samples have different crystalline structures.

A detailed examination of Figure 1a indicates that the LS10 sample has the most diverse morphology, which is characterized by the highest portion of the surface defects. On the other hand, Figure 1d clearly shows that the LS21 sample has the most uniformed morphology with the lowest portion of the surface defects. These defects can serve as active sites (i.e., centers) for the materials’ degradation. Opposingly, a less porous and a more uniform morphology of the sample LS21 (Figure 1d) implies that this sample has the lowest portion of the active centers; thus, LS21 might be more resistant to the degradation process.

**Figure 2:**
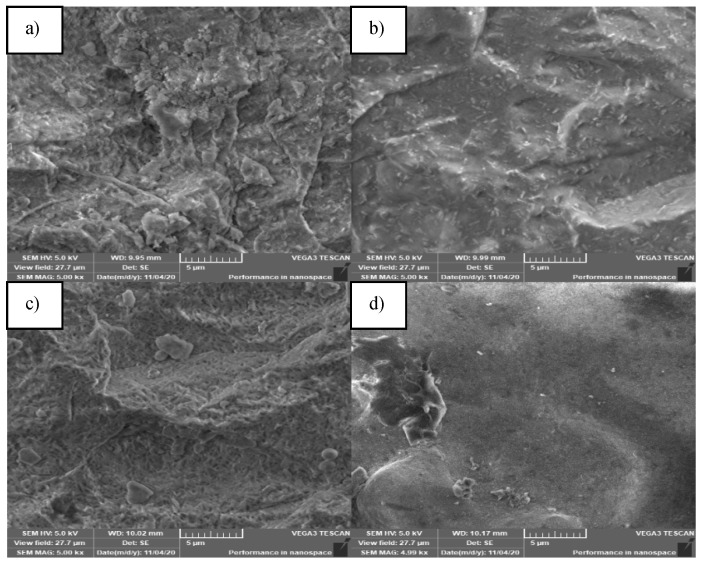
SEM images of the aged samples (**a**) LS10, (**b**) LS11, (**c**) LS20, and (**d**) LS21. Magnification is ×5000.

The samples presented in Figure 1 were aged in 4% acetic acid at 80 °C for 16 h and their SEM images are given in Figure 2. It appears that the aging effect is especially observable in the LS10 and LS20 samples that were not crystallized, whilst this effect is not so evident in the crystallized LS11 and LS21 samples. In the case of LS10 and LS20 (Figure 2a,c), the aging induced a smoother morphology (free of defects), which confirms that the surface defects serve as active centers for aging.

Furthermore, Figure 2a–c clearly indicates that the aged samples LS10, LS11, and LS20 are deformed and full of depressions that are not visible in Figure 1a–c. On the other hand, Figure 2d (vs. Figure 1d) distinctly demonstrates that the LS21 sample is not characterized by depressions, which suggests that the aging in the oral cavity would not perturb long-term interactions between the clinical restoration and the biological environment. The findings in this Section already imply that the LS21 sample is more reliable and durable for the clinical restoration.

### 3.2. XRPD Analysis

**Figure 3:**
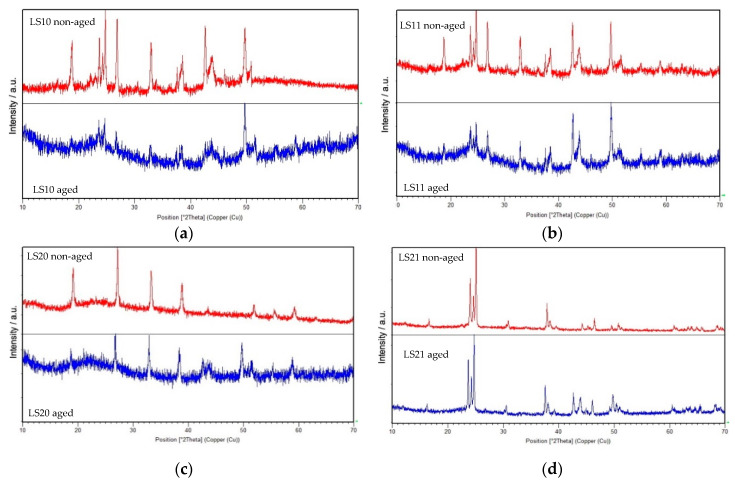
Diffractograms of samples (**a**) LS10, (**b**) LS11, (**c**) LS20, and (**d**) LS21. XRPD patterns colored in red are non-aged samples, whereas patterns colored in blue correspond to samples aged in the acetic acid.

The SEM study showed diverse samples’ porosity and morphology (Figure 1) and as a result, both crystallization and aging effects were investigated by XRPD. Figure 3 displays XRPD patterns colored in red of non-aged LS10 and LS20 (Figure 3a,c) and crystallized LS11 and LS21 samples (Figure 3b,d). According to Figure 3b,d (vs. Figure 3a,c), the sintering process increased crystallinity and decreased porosity, all of which corresponds to a more uniform morphology detected by SEM (Figure 1b,d). Figure 3d also implies that the sample LS21 is of the highest crystallinity and consequently, it should have the lowest portion of the structural defects. The LS2 manufacturer clearly states that the LS2 product has to be crystallized prior to the application [33], which explains a higher crystallinity of the sample LS21 (vs. LS20).

The impact of aging is presented by XRPD patterns colored in blue (Figure 3). It appears that the aging increased the portion of the amorphous phase in the samples LS10, LS11, and LS20 (Figure 3a–c). This is not a coincidence as, according to one study [34], aging in 4% acetic acid for 16 h is equivalent to the degradation of dental ceramics during the course of 2–3 years in the oral cavity.

**Figure 4:**
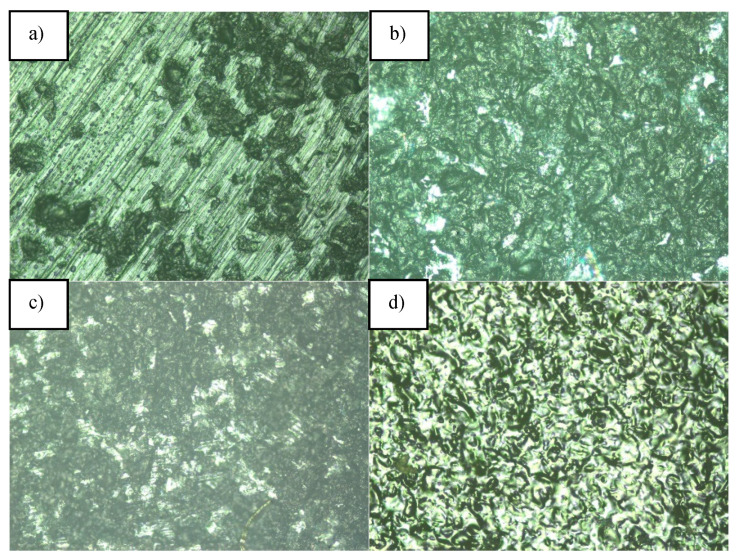
Optical images (×100) of the non-aged samples (**a**) LS10, (**b**) LS11, (**c**) LS20, and (**d**) LS21. Material processing is visible only for LS10 (**a**).

Furthermore, there are several observations that should be commented on. First, the portion of the amorphous phase is especially increased (due to aging) in the case of the sample LS10 (Figure 3a), which had the highest portion of the surface defects prior to aging. Therefore, it can be said that these defects and the porous morphology facilitate both aging and degradation processes. Second, it appears that the LS11 (vs. LS10) was less affected by the treatment in acetic acid, which suggests that the LS1 sample should be crystallized prior to the application. Please note that according to the manufacturer’s instructions, the LS1 can be applied without pre-treatment. Moreover, microscope images of the sample LS10 clearly reveal signs of material processing (Figure 4a) by CAD/CAM technology, which suggests that it might be reasonable to sinter the LS1 product prior to usage after all. On the other hand, LS20 and LS21 samples (Figure 4c,d) do not show any signs of processing.

Finally, the amount of an amorphous phase in LS21 is basically unchanged by aging (Figure 3d), possibly due to the lowest portion of the active centers and surface defects (see Figure 1d and Figure 2d). XRPD study in this work also disclosed that the aging process yielded new phases, especially in the LS21 sample. The impact of aging and crystallization can also be monitored by a size-strain analysis [35,36]. However, both phase identification and the size-strain analysis are currently beyond the scope of this manuscript.

### 3.3. Electrical Conductivity

**Figure 5:**
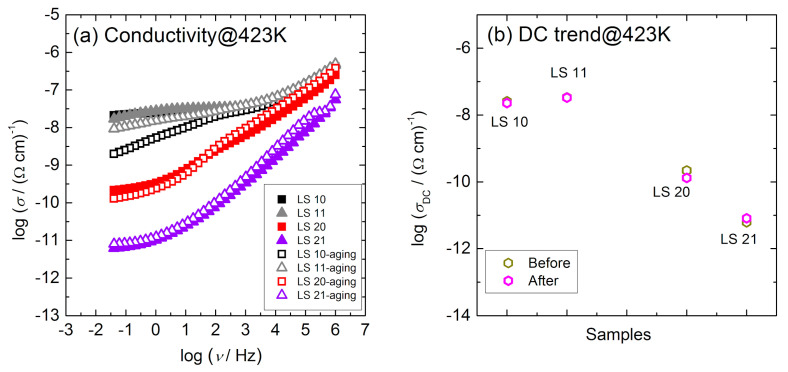
(**a**) Conductivity isotherms and (**b**) DC conductivity of the samples (LS10, LS11, LS20, and LS21) before and after aging obtained at 423 K.

Figure 5 displays conductivity isotherms of the samples LS10, LS11, LS20, and LS21 collected at 423 K in a wide frequency window (10^−2^ to 10^6^ Hz). Characteristically, each isotherm exhibits two features, a plateau at a low frequency that corresponds to the DC conductivity and a dispersion at higher frequencies (Figure 5a). The dispersive behavior is more visible for samples LS20 and LS21, whereas for samples LS10 and LS11, the dispersion is observed only at higher frequencies. In general, conductivity dispersion is a characteristic found in a wide range of materials. Hence, it is not connected to a type of charge carrier [37,38,39,40], but is closely connected to structural disorder. In addition to the two observed spectral features, samples LS10 and LS11 exhibit a pronounced decrease in conductivity in the low-frequency region, as shown in Figure 5a. This behavior is associated with the electrode polarization effect due to the accumulation of mobile ions at the blocking metallic electrode [38,40].

**Scheme 1:**
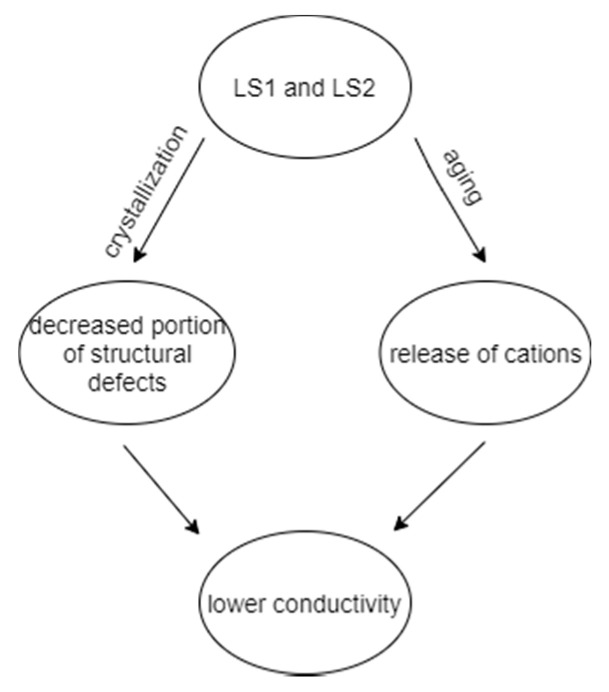
Schematic representation of the impact of both crystallization and aging on the conductivity in the low frequency region of LS1 and LS2 samples in this work.

The DC conductivity of the samples is in the range of 10^−7^ to 10^−11^ S cm^−1^ (Figure 5b), which can be compared to conductivity (10^−7^ S cm^−1^) of LiO_2_-SiO_2_ glasses [6]. Furthermore, the conductivity of the non-aged samples is decreasing in the following trend: LS10 ≈ LS11 > LS20 > LS21. Please note that the portion of the structural defects (Figure 3) in the aforementioned samples also demonstrates the same trend. Therefore, the lower DC conductivity of the samples can be explained by a lower portion of the structural defects (e.g., non-binding oxygen) that hinder the cation diffusion (Scheme 1).

Moreover, the aging process decreased the conductivity in the low frequency region of the LS10, LS11, and LS20 samples (Figure 5a). However, only the most porous LS10 sample (see Figure 1a) showed a straight line in the low frequency region. The straight line suggests the presence of the Warburg [41], which ambiguously indicates the cation(s) diffusion within glass ceramics. It can be concluded that the release of cations (during the aging) from the LS10, LS11, and LS20 samples is the reason for the lower conductivity in the low frequency region (Scheme 1). On the other hand, only the conductivity of the LS21 sample after aging remained almost unchanged, which implies that the loss of the cations might be hindered due to the formation of a new phase. This statement is supported by the XRPD measurement (Figure 3d), which clearly suggests the presence of the new phase(s) after the aging of the LS21 sample.

The conductivity data (Figure 5) were obtained from the impedance spectroscopy (IS) measurements. One should bear in mind that IS data can be studied by the application of the electrical equivalent circuit (EEC) model [42,43,44]. Another additional approach is to extract the distribution function of relaxation times (DRT) [45] from IS data. However, both DRT and EEC analyses were not a primary goal in this research.

Overall, the conductivity (Figure 5), XRPD (Figure 3) and FE-SEM (Figure 1) studies of non-aged samples clearly indicate that the samples’ conductivity in the low frequency region is the lowest in the case of the most crystalline and the less porous LS21 sample, probably due to the lowest portion of the structural defects (e.g., non-binding oxygen). However, the lower conductivity of the aged LS10, LS11, and L20 samples can be explained by the cation loss due to the acetic acid treatment. The release of cations is a common drawback of dental materials and we have previously investigated this issue by aging diverse samples in 4% acetic acid [28]. As LS21 conductivity was not decreased, it can be suggested that there was no loss of the cations, which confirms that this sample is the most durable one.

### 3.4. Limitation of This Study

The samples in this study were artificially aged under in vitro conditions; and thus, the mechanical damage from ageing in the oral cavity was not taken into account. However, the procedures conducted in this study can be applied to glass ceramic materials that are aged in vivo. Although such a tempting task would require an additional modification of the given procedures due to, e.g., differences in a sample dimension.

Furthermore, the exact composition of dental materials is not always given by manufacturers, which hinders proper structural and conductivity analyses. In order to overcome this drawback, such a study should be conducted by using materials of a known composition which mimic the glass ceramic materials (e.g., LS1 and LS2) applied in dental restoration.

### 3.5. The Future Tasks

Herein, the dental ceramics were jointly studied by structural and conductivity techniques. This study also indicates that a plethora of different approaches (e.g., the size–strain analysis, EEC, and DRT) can be applied to study diverse dental ceramics. Furthermore, as the impedance response of dental ceramics can be governed by diffusion, they can also be investigated by the distribution of diffusion times (DDT) [46,47]. Therefore, our future aim is to apply the aforementioned techniques in the study of the dental materials with a special focus on understanding the impact of ageing on both structure and conductivity.

## 4. Conclusions

In this study, zirconia-reinforced lithium-silicate glass ceramics and lithium-silicate glass ceramics were investigated. The samples were tested as-received (LS10 and LS20) and after crystallization (LS11 and LS21) and all of them were aged in 4% acetic acid.

According to SEM images, the LS10 and LS20 samples are characterized by a large number of surface defects (i.e., grains, tubes, and fibers), whilst these defects are not observable in the LS11 and LS21 samples. After the ageing in 4% acetic acid, the surface defects in the LS10 and LS11 samples were not observed. However, except in the LS21 sample, this treatment induced large depressions in LS10, LS11, and LS20 samples. The absence of depressions in the LS21 sample indicates that this sample can be applied for a clinical restoration, as these depressions can induce surface roughness and possible tooth wear of antagonistic teeth. On the other hand, the depressions in LS10, LS11, and LS20 might obstruct the sample’s application in clinical restoration.

The portion of structural defects (e.g., non-binding oxygen) in investigated samples was monitored by XRPD. It was found that the crystalized LS21 sample has the lowest portion of the structural defects. The ageing in the acid increased the fraction of the amorphous phase in the samples LS10, LS11, and LS20, whereas this phase remained unchanged in the case of the LS21 sample.

The findings in this work clearly indicate that a higher conductivity of the samples can be explained by facilitated ion diffusion due to a larger portion of the structural defects. However, after the ageing, the conductivity of the LS10, LS11, and LS20 samples was additionally decreased, which was explained by the release of cations from the samples. Since LS21 conductivity remained almost unchanged after the ageing, it was concluded that this sample has the highest chemical durability.

To summarize, herein, an experimental setup was proposed in order to monitor the ageing properties of the dental ceramic. According to the findings presented in this study, only the morphology and conductivity of the LS21 sample were practically unchanged by the ageing, which makes LS21 a reliable material for a clinical restoration. The results in this study clearly show that the crystallized LS2 material has demonstrated the best ageing properties.
